# Downregulation of claudin-7 potentiates cellular proliferation and invasion in endometrial cancer

**DOI:** 10.3892/ol.2013.1330

**Published:** 2013-05-08

**Authors:** XIAOCUI LI, YUHONG LI, HAIFENG QIU, YUDONG WANG

**Affiliations:** 1 Department of Gynecology, International Peace Maternity and Child Health Hospital, Shanghai Jiaotong University, Shanghai 200030, P.R. China; 2 Department of Obstetrics and Gynecology, First People’s Hospital, Shanghai Jiaotong University, Shanghai 200030, P.R. China

**Keywords:** claudin-7, invasion, endometrial cancer

## Abstract

Claudin-7, a tight junction protein, has been demonstrated to be abnormally regulated in several types of human cancer. The present study aimed to investigate the expression and function of claudin-7 in endometrial cancer. In total, 31 pairs of endometrial cancer samples and their adjacent normal tissues were used to detect the expression of claudin-7 by immunohistochemical staining. Compared with the corresponding normal tissues, 45.2% of the endometrial cancer tissues exhibited weak or absent claudin-7 protein expression. Low levels of claudin-7 were correlated with a late tumor stage (P=0.023) and low histological grade (P=0.018). Claudin-7 was either overexpressed in AN3CA endometrial cancer cells, via plasmid cDNA transfection, or silenced by RNA interference in Ishikawa cells. Following either type of experimental manipulation, cellular proliferation and invasion were determined by 3-(4,5-dimethylthiazol-2-yl)-2,5-diphenyltetrazolium bromide (MTT) assay, wound healing and transwell assays, respectively. The silencing of claudin-7 significantly increased cellular proliferation (P=0.032) and invasion (P=0.020) rates. Consistent with these results, the increased expression of claudin-7 decreased the proliferation (P=0.021) and invasiveness (P=0.012) of the AN3CA cells. A low expression of claudin-7 in the endometrial cancer cells was indicative of a late tumor stage and low histological grade. Additionaly, restoration of claudin-7 inhibited the proliferation and invasion of endometrial cancer cells, thus providing a potential therapeutic strategy.

## Introduction

The loss of cell-cell adhesion is a significant step in tumor cell metastasis (1). Claudins are a family of tight junction proteins that regulate cell adhesion and polarity. Thus, they appear to be significant in regulating the process of metastasis. First described in 1998, claudins contain four transmembrane domains and, to date, 24 family members have been identified. These proteins have molecular weights of 20–27 kDa and are widely expressed in the majority of epithelial cells (2,3). Certain claudin family members have been identified to be abnormally regulated in several types of cancer in humans. In particular, claudin-7 was observed to be downregulated in breast cancer and head and neck squamous cell carcinoma. These findings suggest that claudin-7 may be involved in the epithelial-mesenchymal transition (EMT). Following the restoration of claudin-7 levels, cancer cells have been demonstrated to exhibit decreased motility and invasion abilities (4,5).

Endometrial cancer is the primary gynecological malignancy in the majority of countries (6). In the USA, ∼8,010 fatalities resulting from endometrial cancer and 47,130 new cases are predicted this year (6–8). At the time of diagnosis, ∼25% of patients present with regional or distant metastases (stages III or IV). Notably, the prognosis associated with this group is usually unfavorable (9). Therefore, specific strategies targeting tumor invasion should be a priority. However, detailed mechanisms governing endometrial cancer metastasis are not well defined. Previous studies have suggested that claudins may play a critical role in this process.

In endometrial cancer, several of the claudins, including claudin-1, -3 and -4, have previously been demonstrated to be involved in maintaining tight junctions and in preventing tumor cell dispersion and invasion (10,11). In the present study, the functions of claudin-7 were investigated in endometrial cancer.

## Materials and methods

### Immunohistochemical staining assay

Human endometrial cancer tissue microarrays, comprising 31 pairs of endometrial cancer tissues and their corresponding normal endometrial tissues, were purchased from Shanghai Xinchao Biotechnology (Shanghai, China). All tissues had been acquired via surgical resection. The cancer cases were classified and graded according to the criteria of the International Federation of Obstetrics and Gynecology (FIGO, 2009). This study was approved by the ethics committee of the International Peace Maternity Child Health Hospital, Shanghai Jiaotong University. In brief, the tissue sample slides were rehydrated and antigen retrieval was performed in the microwave with ethylenediaminetetraacetic acid (EDTA; pH 8.0). The slides were then incubated with primary anti-claudin-7 antibodies (1:1,000; Epitomics, Inc., Burlingame, CA, USA) overnight at 4°C. Antibody staining was visualized with 3,3′-diaminobenzidine (DAB; Invitrogen Life Technologies, Carlsbad, CA, USA). For evaluation, the following criteria were used: 0, no expression (complete negative staining); 1, weak expression (1–15% positive staining); 2, moderate expression (16–49% positive staining); and 3, strong expression (50–100% positive staining). Scores of 0 and 1 were defined as negative expression. All slides were independently scored by two pathologists.

### Cancer cell lines and cultures

The RL95-2, Ishikawa, AN3CA and KLE endometrial cancer cell lines, were routinely cultured in Dulbecco’s modified Eagle’s medium (DMEM)/F12 (Gibco, Auckland, New Zealand) supplemented with 10% fetal bovine serum (Biowest, Nuaillé, France) at 37°C with 5% CO_2_.

### RNA extraction and quantitative real-time reverse transcription polymerase chain reaction (RT-PCR)

Total RNA was extracted using TRIzol reagent (Invitrogen Life Technologies) and reverse transcribed with the RT kit from Takara Biotechnology Co., Ltd. (Dalian, China). The primers used for claudin-7 were as follows: Forward, 5′-AGAG CACG G G GATGATGAG-3′ a nd reverse, 5′-CACCCATGGCTATACGGGC-3′. The PCR conditions were as follows: 95°C for 30 sec, 35 cycles at 95°C for 5 sec, then 60°C for 30 sec. β-actin was used as an endogenous control. The relative mRNA expression was calculated using the 2^−ΔΔCt^ comparative CT method.

### Western blot analysis

The primary antibodies for claudin-7 were obtained from Epitomics, Inc. The mouse monoclonal anti-glyceraldehyde-3-phosphate dehydrogenase (GAPDH), horseradish peroxidase (HRP)-conjugated anti-rabbit and anti-mouse secondary antibodies were purchased from Boshide Biotechnology Company (Wuhan, China). The specific bands were developed with enhanced chemiluminescence (Beyotime, Shanghai, China).

### Silencing claudin-7 in Ishikawa cells

Claudin-7-specific siRNA was purchased from Shanghai Genepharma Co., Ltd. (China). The cells were cultured in 6-well plates for 24 h prior to being transfected with Lipofectamine 2000 (Invitrogen Life Technologies), according to the manufacturer’s instructions.

### Overexpressing claudin-7 in AN3CA cells

The plasmid pcDNA3.1-claudin-7 was purchased from Genearray Biotech Company (Shanghai, China) and verified by sequence analysis. The AN3CA cells were transfected with pcDNA3.1-claudin-7 or an empty vector using Lipofectamine 2000. Subsequently, real-time PCR and western blot analysis were performed to verify the changes in claudin-7 expression.

### Proliferation assay

The proliferation of the Ishikawa and AN3CA cells was determined by 3-(4,5-dimethylthiazol-2-yl)-2,5-diphenyltetrazolium bromide (MTT) assay. Briefly, 4×10^3^ cells were seeded in each well of a 96-well plate and incubated overnight. At the appropriate time (48 and 72 h), the cells were incubated with 10 *μ*l MTT (5 mg/ml; Sigma, St. Louis, MO, USA) for 4 h. Formazan crystals were subsequently dissolved in 100 *μ*l dimethylsulfoxide (DMSO; Sigma). The absorbance of the solution was measured at 570 nm. All measurements were repeated in triplicate.

### Transwell assay

The transwell chamber system (Applied Biosystems, Foster City, CA, USA) was used to investigate cellular invasive abilities. Briefly, 1×10^4^ Ishikawa or AN3CA cells were seeded into the upper chamber. Subsequently, 500 *μ*l conditioned medium was added to the bottom chamber. Following 24 h of incubation, the cells that had migrated to the bottom membrane were stained and counted under a microscope. This experiment was performed in triplicate.

### Statistical analysis

P<0.05 was considered to indicate a statistically significant difference. SPSS software, version 16.0, (SPSS Inc., Chicago, IL, USA) was used for the statistical analysis. The analysis was performed using either a χ^2^ test or a t-test.

## Results

### Claudin-7 expression in human endometrial cancer

**Figure 1.**
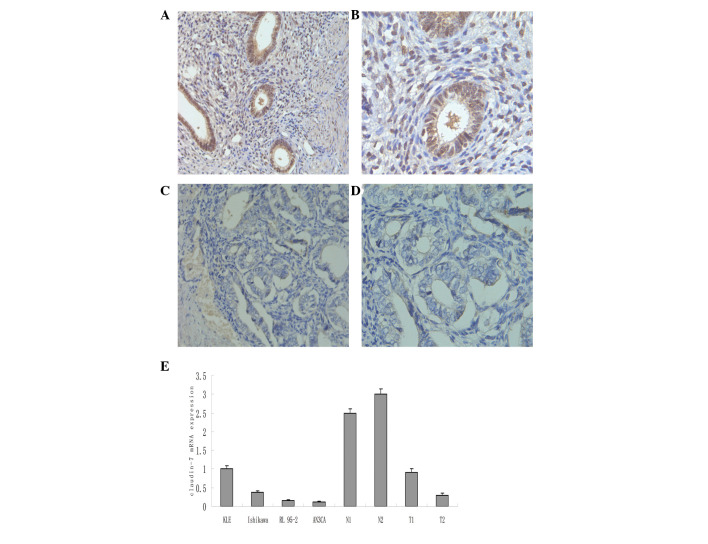
Immunohistochemical staining of claudin-7 expression in (A and B) normal endometrium and (C and D) cancer tissues. Original magnification, (A and C) ×200 and (B and D) ×400; staining, DAB. (E) Claudin-7 mRNA expression in endometrial cancer cell lines, as detected by real-time reverse trancription-polymerase chain reaction analysis. N, normal tissues; T, tumor tissues; DAB, 3,3′-diaminobenzidine.

Compared with the corresponding normal endometrial tissues, the endometrial cancer tissues exhibited significantly downregulated claudin-7 expression (Fig. 1, P<0.01). In the cancer tissues, approximately half of the patients (14/31, 45.2%) demonstrated a loss or the total negative expression of claudin-7. A real-time RT-PCR analysis of the KLE, RL 95-2 and Ishikawa cells revealed that these cell lines positively expressed claudin-7, and that by contrast, the AN3CA cells were claudin-7-negative (Fig. 1).

### Correlations between claudin-7 expression and clinicopathological characteristics

According to the statistical analysis, the reduced expression of claudin-7 was significantly correlated with the tumor stage (P=0.023) and histological grade (P=0.018), but not with the remaining characteristics that were examined (Table I).

### Correlations between claudin-7 expression and other immunohistochemical parameters

No correlation was observed between claudin-7 expression and ER, PR or p53 expression (P=0.125, P=0.318 and P=0.266, respectively; Table I).

### Claudin-7 silencing increases the proliferation and invasion of Ishikawa cells

**Figure 2.**
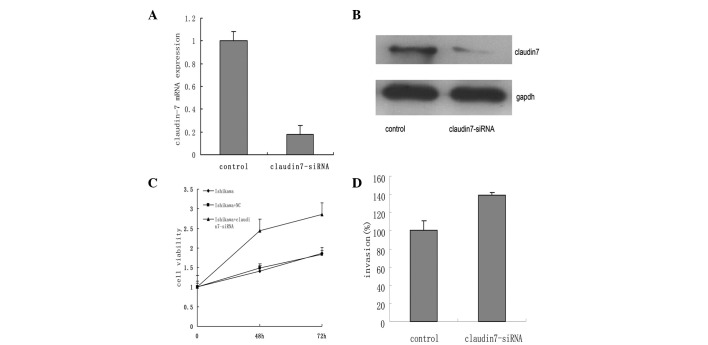
Downregulation of claudin-7 in Ishikawa cells and its effect on cellular growth and invasion. (A) mRNA and (B) protein expression of claudin-7 were reduced following RNA-interference. In Ishikawa cells with decreased claudin-7, cellular (C) growth and (D) invasion were all increased. NC, negative control.

RNA interference resulted in an ∼90% knockdown of claudin-7 mRNA levels in the Ishikawa cells (Fig. 2A and B). The MTT assay revealed that the Ishikawa cells treated with claudin-7-siRNA possessed a higher growth rate compared with the untreated and empty vector (negative control) Ishikawa cells (P=0.032; Fig. 2C). In the transwell assay, the claudin-7-silenced Ishikawa cells were more invasive than the control group (P=0.02; Fig. 2D).

### Claudin-7 overexpression decreases the tumorigenic properties of AN3CA cells

**Figure 3.**
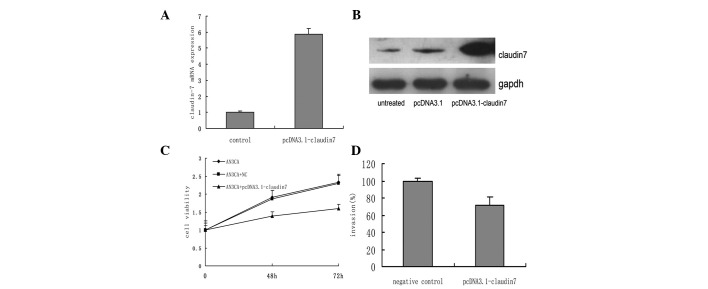
Upregulation of claudin-7 in AN3CA cells and its effect on cellular growth and invasion. (A) mRNA and (B) protein expression of claudin-7 was upregulated following transfection with pcDNA3.1-claudin-7. Cellular (C) growth and (D) invasion were impaired by claudin-7 overexpression. NC, negative control.

The transfection of the AN3CA cells with pcDNA3.1-claudin-7 resulted in the upregulation of claudin-7 at the mRNA and protein levels (Fig. 3A and B). Cellular proliferation and invasion were significantly suppressed following transfection (P=0.021 and P=0.012, Fig. 3C and D, respectively), compared with the untreated and empty vector (negative control) AN3CA cells.

## Discussion

In numerous types of human cancer, the number of cell-cell junctions decreases, permitting the escape of cancer cells from their primary sites, along with the acquisition of invasive and metastatic properties (12,13). Therefore, targeting cell-cell junctions may be a valid strategy for the treatment of cancer. A number of claudins, including claudin-7, have been identified to be downregulated in various types of human cancer. Claudin-7 has been shown to be downregulated in head and neck squamous cell carcinoma, invasive ductal breast cancer and invasive esophageal cancer (4,5,14). However, these studies did not analyze the manner in which claudin-7 functions to inhibit cancer cells from undergoing invasion and metastasis. Thus, the present study investigated the expression and potential mechanisms governing claudin-7 in endometrial cancer.

The study observed that claudin-7 was frequently down-regulated in human endometrial cancer tissues. To investigate the mechanism by which claudin-7 affects endometrial cancer cells, claudin-7-specific siRNA was first transfected into Ishikawa cells (those demonstrating normal claudin-7 expression). The results demonstrated that invasion was significantly upregulated in the Ishikawa cells. Cellular proliferation was also significantly upregulated. Full-length claudin-7 cDNA was then cloned and overexpressed in the AN3CA cells (which were claudin-7-negative), and MTT and invasion assays confirmed similar effects.

It has been suggested that the effects of claudin-7 were generated through the attenuated activation of MAPK/ERK signaling (14). The interactions between the MAPK pathway and tight junction proteins have previously been identified. For example, the tight junction membrane protein, occludin, participates in the activation of the MAPK signaling pathway (15). In the hepatic cell lines derived from occludin-deficient mice, MAPK activation was revealed to be downregulated, triggering apoptosis and increasing claudin-2 expression. The PI3K/Akt pathway may also interact with tight junction proteins. It has been also demonstrated, in these hepatic cell lines, that the activation of Akt is decreased, while cell apoptosis is increased (16).

Overall, the present study demonstrated that claudin-7 is frequently downregulated in endometrial cancer, and that this is correlated with the tumor stage and histological grade. The ectopic expression of claudin-7 significantly regulates the proliferation and invasion of endometrial cancer cells. Therefore, these findings may provide a potential therapeutic target for the treatment of endometrial cancer.

## Figures and Tables

**Table I.** Correlations between claudin-7 expression and clinicopathological characteristics in 31 endometrial carcinoma samples.

Variables	No. of patients	Claudin-7 expression		P-value^a^
		Negative	Positive	
Age (years)				
<60	17	7	10	0.715
≥60	14	7	7	
Stage				
I	21	9	12	0.023
II	5	2	3	
III	4	2	2	
IV	1	1	0	
Grade				
1	23	8	15	0.018
2	5	3	2	
3	3	3	0	
Myometrial invasion				
<1/2	19	9	10	0.560
≥1/2	12	5	7	
ER expression				
Negative	9	5	4	0.125
Positive	22	10	12	
PR expression				
Negative	5	2	3	0.318
Positive	26	12	14	
p53 expression				
Negative	23	10	13	0.266
Positive	8	3	5	

a From the χ^2^ test.
